# A Novel Approach for Objective Assessment of White Blood Cells Using Computational Vision Algorithms

**DOI:** 10.1155/2018/4716370

**Published:** 2018-11-13

**Authors:** Cesar Mauricio Rodríguez Barrero, Lyle Alberto Romero Gabalan, Edgar Eduardo Roa Guerrero

**Affiliations:** ^1^KINESTASIS Seedlings of Research, University of Cundinamarca, Fusagasugá, Colombia; ^2^GITEINCO Research Group, University of Cundinamarca, Fusagasugá, Colombia

## Abstract

In the field of medicine, the analysis of blood is one of the most important exams to determine the physiological state of a patient. In the analysis of the blood sample, an important process is the counting and classification of white blood cells, which is done manually, being an exhaustive, subjective, and error-prone activity due to the physical fatigue that generates the professional because it is a method that consumes long laxes of time. The purpose of the research was to develop a system to identify and classify blood cells, by the implementation of the networks of Gaussian radial base functions (RBFN) for the extraction of its nucleus and subsequently their classification through the morphological characteristics, its color, and the distance between objects. Finally, the results obtained with the validation through the coefficient of determination showed an overall accuracy of 97.9% in the classification of the white blood cells per individual, while the precision in the classification by type of cell evidenced results in 93.4% for lymphocytes, 97.37% for monocytes, 79.5% for neutrophils, 73.07% for eosinophils, and a 100% in basophils with respect to the professional. In this way, the proposed system becomes a reliable technological support that contributes to the improvement of the analysis for identification of blood cells and therefore would benefit the low-level hematology establishments as well as to the processes of research in the area of medicine.

## 1. Introduction

The complete blood count (CBC) is a test that provides information relevant to the pathologic diagnosis of a patient; by the analysis the professionals in hematology observe the sample and make cell counts with the purpose to find morphological alterations of interest [1]. Within the analysis of the leucogram or count of leukocytes (white blood cells), this procedure is to determine the number of white blood cells in a given unit of blood volume of total and differential form. This test it is essential to know the values of concentration and morphology of blood cells, providing information associated with a wide range of diseases that are present in the body [2] as the estimate of malaria parasites [3].

At present, the hematology laboratories and centers of high level carried out a large number of analyses of blood per day, using automated techniques [4], in order to reduce the amount of time at each examination. However, this technique requires sophisticated and expensive equipment with technology imported from difficult access to low-level hematology establishments, as well as requiring the constant calibration by specialists [5]. For this reason, health centers or hematologists opt to perform the analysis manually, incorporating high levels of subjectivity and measurement error.

In recent years research in the field of computer vision has been developed, which has made it possible to remove and recognize patterns or objects in images little perceptible to the human vision. In [6] an efficient method for the automatic segmentation through the use of active contour techniques is posed. On the other hand, in [7] neural networks are used for the classification of the white blood cells in a blood smear. Other authors in [8] propose the segmentation of the nucleus in the white blood cell through the use of automatic thresholding classical techniques as OTSU [9, 10]. In [11] an iterative method of region growing is applied, to get better segmentation in images that have degeneration in the sample. Although in Colombia there is no presence of commercial systems, there is already significant progress in that present research [12].

The purpose of this research was to develop a system for the identification and classification of white blood cells, which decreases the subjective errors in the manual analysis through the development of a system that uses image processing techniques, in order to provide technological support reliable with repeatability in the results.

For its development, the acquisition of images from the peripheral blood smears was made, which are adapted using the YCbCr color space and in this way extract the regions belonging to the white blood cell count using the technique of the networks of Gaussian radial base functions; later a graphical interface to assist the professional in hematology during the procedure of identification of white blood cells was designed. Finally, the results of the classification of the white blood cells with respect to the manual analysis were validated.

In the present research work, we present the results of the development, implementation, and validation of a computational tool for the classification of blood cells from microscopic images of blood smears obtained from 13 different individuals.

## 2. Materials and Methods

In the first place, the preparation of blood samples was carried in accordance with the protocols laid down in the manual of clinical rationale in hematology [13], which define a series of steps for the preparation of the smears of blood samples, then the blood is drawn by a venous puncture; later a drop of blood is placed on a microscope slide and the spread of the same. Subsequently, it applies the dye type Wright in order to differentiate between white blood cells with respect to the substance of the sample. Finally, using a binocular microscope LEICA DM500 with digital camera led WiFi model ICC50W, later the capture made 260 microscopic images in JPG format with a resolution of 1200x1600, from a set of 13 blood smears of different individuals, previously analyzed by specialists in a specialized blood center in the city of Fusagasugá, Colombia.

**Figure 1:**
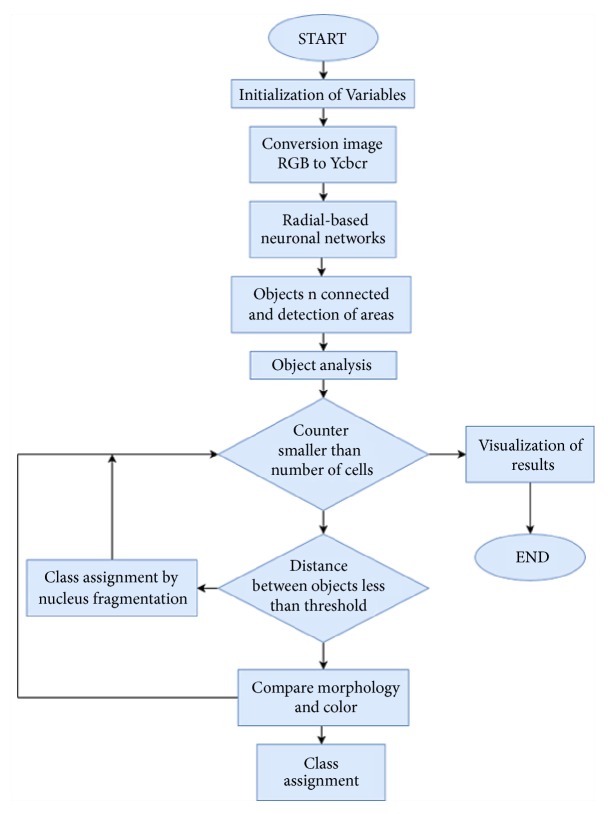
Proposed methodology.

The methodology proposed for the identification of blood cells is divided into 4 stages, with different processes as shown in Figure 1. At the stage of preprocessing the image RGB is converted to YCbCr color space for an improvement in the contrast of the nuclei of cells with respect to the background. In the stage of segmentation they are extracted from the nuclei of cells using the technique of the networks of Gaussian radial base functions (RBFN). After the extraction process, in the third stage the classification was made according to the morphological characteristics and color present in the cells. Later, in the fourth stage the results obtained with the system in comparison with specialists in hematology are validated. Finally, through a graphical user interface, the results obtained in the identification are displayed. The following describes each of the techniques implemented in the stages described above.

### 2.1. Preprocessing of Images

Once the microscopic images are obtained from the blood smears, they are converted from the RGB color space to YCbCr [14], which allows better highlighting the nuclei of the globules present in the image with respect to other color spaces.

### 2.2. Networks of Gaussian Radial Base Functions

For the extraction of the white blood cells the average value of the pixels present in the nucleus of the cell obtained, according to a database of 260 images of 13 individuals. Following this, the criterion of the Euclidean distance using equation 1 is applied, in order to represent the difference between each pixel value in the image, with respect to the pixel average found in the database.

(1) $$\begin{array}{c} D=\sqrt{{\left(R_{i}-R_{pm}\right)}^{2}+{\left(G_{i}-G_{pm}\right)}^{2}+\left(B_{i}-B_{pm}\right)} \end{array}$$

where Ri, Gi, and Bi represent the levels of red, green, and blue color of each pixel in the image and Rpm, gpm, and bpm represent the levels of red, green, and blue color of the pixel average previously obtained of the white blood cells in in the image.

Following this evaluates the distance D in a Gaussian radial basis function [7] for each pixel using (2), where *α* is the number of values that each pixel can have in this case 255. This provides a value between 0 to 1, where 0 indicates that the pixel analyzed does not look like the average pixel belonging to the object, while 1 indicates that they are identical; in this way the image is thresholdized through the use of a bell curve [15].

(2) $$\begin{array}{c} E=e^{-D/\alpha } \end{array}$$

In this way we take the values with a very low coefficient of deviation with respect to the central value of the neuron in the campaign, in order to segment the objects of interest in this case, the nuclei of the white blood cells with greater accuracy.

After obtaining the thresholded image, the method of objects (n) connected was applied to determine whether two pixels are adjacent to each other in a binary image [14]. In the image each group of pixels connected corresponds to the nuclei of the cells, because platelets present in the images which have pixel values very similar to those of the nuclei are also removed. To do this, morphological operations were used in order to eliminate small areas belonging to unwanted objects in the image.

### 2.3. Morphological Descriptors

To measure the properties of the objects morphological descriptors were used which allowed finding a relationship between shape and size to perform the classification [14]. Among the measures that stand out the eccentricity, solidity, elongation, among others were found. In addition, they allowed knowing the centroid or coordinates of the nucleus center of the cell on the image. According to the different measurements, an analysis of the morphological characteristics of each cell type was carried out, in order to find the characteristic that would allow differentiating the classes of globules, with the solidity being the characteristic with the most variation between each type of cell. This feature made it possible to differentiate the nucleus of the cell as follows: if the object segmented is compact, its value is close to 1 and in the case that the object is not compact its value is close to 0.

In addition of the use of morphological descriptors in the classification, A measurement of the color belonging to the nucleus and cytoplasm of the cell was also implemented, in order to make the system more robust at the time of classifying, allowing differentiating monocytes and eosinophils, which are two types of cells that have very similar characteristics in the shape of the nucleus but with variation of color.

### 2.4. Distance between Objects

It consists of finding a distance value between two coordinates that represent two objects found in the image. This is done by a mathematical process based on the Pythagorean theorem equation (3) where the value of the hypotenuse is interpreted as distance.

(3) $$\begin{array}{c} c^{2}=a^{2}+b^{2} \end{array}$$

To apply this concept, you must know the coordinates of the center of the object in the image; once these values are obtained, a right triangle is drawn between these two points in order to apply the Pythagorean theorem and determine the separation between the objects. In the system, this distance is necessary to determine if it is a fragmented nucleus, since neutrophils can be found in embedded or fragmented. With the value below a certain distance threshold between two objects in the image, the system determines what type of nucleus is present at the time of classifying the cells.

## 3. Results

### 3.1. Image Preprocessing

**Figure 2:**
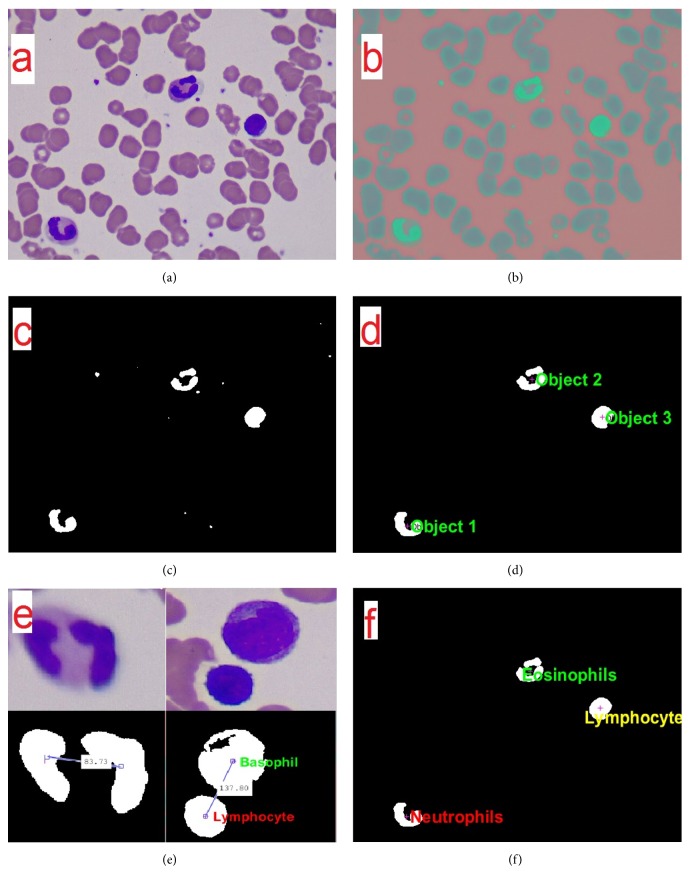
(a) Original Image. (b) Image in YCbCr. (c) Segmentation based on networks of Gaussian radial base functions. (d) Application of morphological operators. (e) Analysis of morphological characteristics. (f) Classification of white blood cells.

The conversion to other color spaces applied to the original image in RGB was carried out in order to see their impact on the definition of the nuclei with respect to the fund to improve the stage of segmentation. It was noted in the YCbCr color space an improvement in the contrast of the nuclei of cells with respect to the background which makes the segmentation by networks of radial basis functions; in Figures 2(a) and 2(b), there is evidence of improvement in the definition of the nuclei with respect to the background.

### 3.2. Segmentation of White Blood Cell

Figure 2(c) shows the process of segmentation of the nuclei of white blood cells, through which its shape and size are preserved. At the same time, small particles were extracted that are present in the blood platelets because they have the same shade of color. The image resulting from the segmentation process contains binary values (1 white and 0 black), which allows the use of morphological operators to remove small particles generated by platelets whose area is less than 1500 pixels for images with a spatial resolution of 1200x1600 pixels, because a nucleus of a cell presents major areas to this value. Followed this an operator of dilatation with a structuring element of type disk with a radius of 3 pixels was implemented, and finally it fills in gaps of the nuclei in order to give uniformity as may be evidenced in Figure 2(d).

### 3.3. Classification of White Blood Cell

Once the images are obtained with the objects belonging to the nuclei of the cells, the value of the centroids is found, in order to identify the position of each of these cells in order to subsequently perform an analysis of the morphological characteristics, distance between objects, and the color of the cytoplasm, allowing the classification according to each type of white blood cell. Initially, there was a sweep through the entire image by calculating the values of the morphological descriptors of each object present in it as shown in Figure 2(d).

Subsequently, Table 1 describes the most relevant features that are used by the algorithm in the identification of the cells. Objects 1 and 2 according to the specialists belong to two different types of cells such as neutrophil and eosinophil, but these present values in its morphology very similar making it impossible for their identification by applying only this analysis, so it was necessary to perform an analysis of the tonality of the cytoplasm of the cell as evidenced in Figure 2(e); this analysis was performed by knowing the coordinates of objects in order to determine the values of the different components of color in the image on the periphery of the nucleus of the cell in order to make the correct identification of the cell.

Some white blood cells present fragmented nuclei as shown in Figure 2(e), where a neutrophil with a nucleus divided into two was found. To prevent the algorithm taking objects segmented as two different cells, the calculation is made of the distance of the centroids of each one. The calculation is based on the Pythagorean theorem where the hypotenuse of the triangle formed by the coordinates represents the distance measured in pixels. This measurement between the objects allows determining if these belong to the same cell, if it is below a threshold of 115 pixels away.

Applying a joint analysis of the different methods mentioned above, the algorithm performs the identification of the cells, classifying them in the 5 different populations (neutrophils, basophils, monocytes, eosinophils, and lymphocytes) effectively having a good performance with very similar cell populations, as evidenced in Figure 2(f).

## 4. Discussion

The purpose of the system is to contribute to the identification of white blood cells in a semiautomatic manner, based on the images obtained from the blood smear. This identification was made through an analysis of the different characteristics of each of the cells, in order to achieve a classification in each of their populations. The validation was carried out by comparing the results obtained with the system with respect to the results issued by a specialist in hematology, finding thus a correlation index between the two measurements to corroborate the accuracy of the system; for this purpose 20 images per individual were used.  Table 2 shows the analysis performed by the professional in hematology and by the computational tool, respectively, for the 13 individuals.

**Figure 3:**
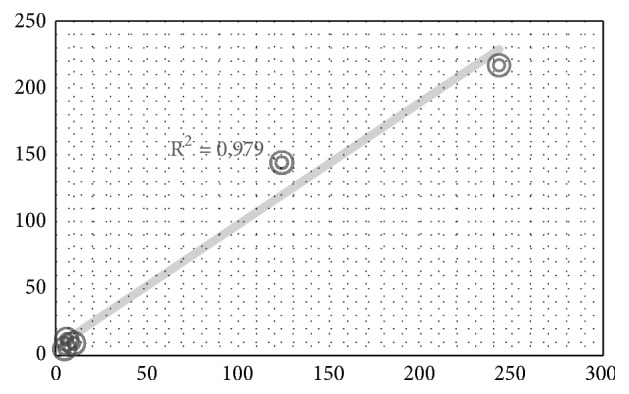
Validation of the tool versus analysis of professional in hematology.

The validation of the results of the system with respect to the analysis carried out by a professional in hematology presented a coefficient of determination R2 = 0.979, as shown in Figure 3, indicating a high correlation between the measurements per individual.

**Figure 4:**
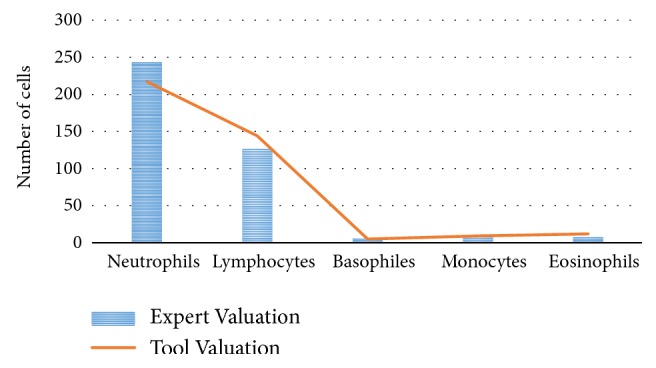
Comparison of results of the identification of white blood cells, carried out by the professional in hematology and the computational tool.

On the other hand, a correlation analysis was made between the hematology professional and the developed tool, in which it was evidenced that the obtained data follow the trend of the results from the analysis of the hematology professional; as shown in Figure 4, it shows the trend of the results obtained by the algorithm against the results obtained by the expert, evidencing high correlation between the two measurements to identify the cells of white blood cells in its 5 different populations. Therefore, it is possible to say that the implementation of this segmentation algorithm based on networks of radial basis functions and classification by morphological characteristics had a good performance in the identification of white blood cells.

On the other hand, the validation of the computational tool with respect to the professional in hematology for each class of white blood cell is present in the blood.  Table 3 shows the percentages of correlation for each class. For the class of basophils, a high percentage of correlation (100%) was presented due to the low number of cells; according to [16], the percentage of basophils in an adult sample is 0.4% compared to the total number of cells in the sample, while for eosinophils it is 2.3%, for monocytes it is 5.3%, for lymphocytes it is 30%, and for neutrophils it is 62%.

**Figure 5:**
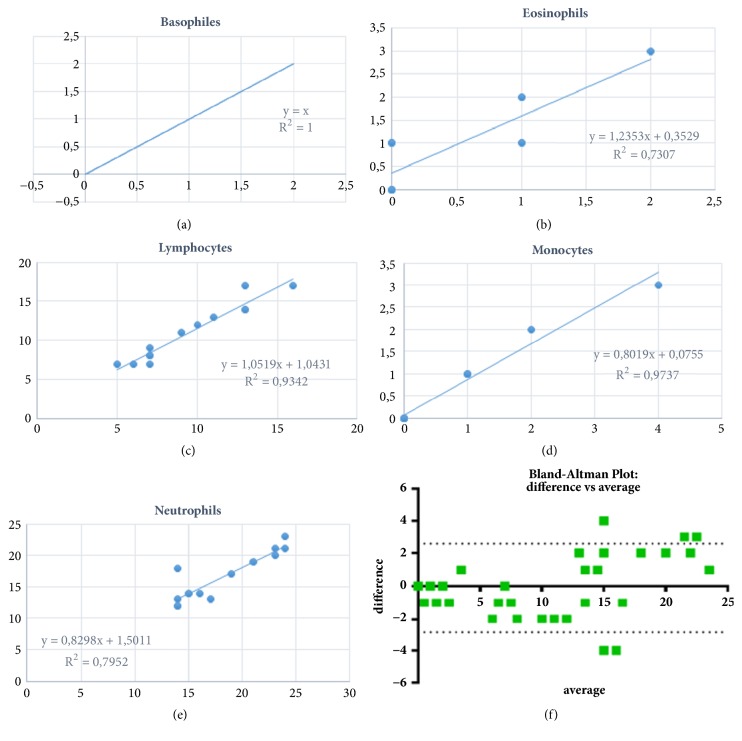
The coefficient of determination for each class of white blood cells. (a) Basophils, (b) eosinophils, (c) lymphocytes, (d) monocytes, (e) neutrophils, and (f) the Bland-Altman test between the professional in hematology and the tool.

The accuracy of the tool to identify the types of cells was also determined, achieving high percentages of success with respect to what was established by the professional in hematology. For basophils it was 100%, while the lowest evidence in the eosinophils has a value of 73.07% as shown in Figures 5(a) and 5(b), respectively, and for lymphocytes a percentage of 93.42% was determined, as shown in Figure 5(c). In the same way a percentage of 97.37% for the cells belonging to the population of monocytes was obtained as seen in Figure 5(d). Finally, for the population of neutrophils a percentage of 79.52% was obtained, as evidenced in the correlation of Figure 5(e).

On the other hand, using the Bland-Altman test which represents the difference between two measurements shown in the Figure 5(f), there was significant variability between the identification of white blood cells by the professional in hematology using the manual technique and by the computational tool developed. The results obtained show the differences with a ratio of 0.923 in a confidence interval of 95% (2.68 to 2.77), indicating that the two measurements were similar. If the two methodologies were the same the expected proportion would be 1. Finally, we compared the accuracy of the results and the characteristics of the system developed with regard to the current semiautomatic systems, as shown in Table 4. The proposed system was implemented in MatLab, obtaining percentages of accuracy higher than 79% indicating that there was no significant variability between the two measurements.

## 5. Conclusions

Research in the area, it demonstrated the importance of the computer vision to automate repetitive processes that are carried out manually through observation, by providing an objective analysis with high precision, in short periods of time.

The process of obtaining blood samples was carried out by means of the protocol stipulated in the manuals for the hematologic analysis, in which the staining plays a significant role since it highlights the white blood cells of other objects, allowing for the correct identification of the white blood cells through images. Although there were problems with some samples during the acquisition of the images, they must take into account the space of the extended you are observing with the objective of the microscope, given that the identification of the white blood cells is carried out in the fields where the cells are not clustered.

The implementation of the networks of Gaussian radial base functions in conjunction with morphological descriptors and the distance between objects yielded better results in the segmentation of the nuclei with regard other segmentation techniques used, due to that highlights and extracts which completely removed the nuclei while retaining its shape on images directly in the color space.

The results obtained in the validation of the tool expose the good performance that was achieved in comparison with the results obtained from the analysis of a specialist. However, when the individual suffers from some types of infectious process or a type of pathology, it causes alterations directly in the white blood cells, thus hindering the precise identification of the cell type. However, the correlation index presented percentages higher than 95% for the individuals; on average the tool presented 98.09% accuracy in the classification of white blood cells with respect to the manual method performed by the professional in hematology.

Finally, the system developed for the analysis of blood cells allowed the technological support to the specialist, with the ability to reduce the errors generated by the subjectivity presented in the interpretation on the part of the specialist and in this way it provides reliable results. In Colombia, this tool would provide an economic alternative accessible for analysis in future research. In addition, it is proposed to expand the study to perform the complete count and classification of white blood cells, in addition to diagnose diseases associated with the reference test ranges, thus contributing to the processes of interdisciplinary research in the University of Cundinamarca.

## Figures and Tables

**Table 1:** Values obtained with the morphological descriptors.

	1 object	2 objects	3 objects
Centroid	X=266	X=866	X=1202
	Y=1054	Y=394	Y=563

Strength	0.778	0.777	0.965

Area	9127	8549	8257

Perimeter	523.361	600.605	340.583

**Table 2:** Analysis of the smears of blood performed by the professional in hematology versus computational tool.

Analysis of professional expert in hematology versus Tool										
**individuals**	**Neutrophil**		**Lymphocyte**		**Basophil**		**Monocyte**		**Eosinophilic**	
	**Expert**	**Tool**	**Expert**	**Tool**	**Expert**	**Tool**	**Expert**	**Tool**	**Expert**	**Tool**
**1**	23	21	13	14	2	2	1	1	2	3
**2**	16	14	7	8	0	0	0	0	0	0
**3**	14	18	13	14	0	0	0	0	1	1
**4**	17	13	13	17	0	0	0	0	1	1
**5**	24	23	16	17	1	1	1	1	0	0
**6**	15	14	7	7	1	1	1	1	1	2
**7**	14	12	10	12	0	0	0	0	0	0
**8**	23	20	9	11	0	0	0	0	0	1
**9**	14	12	7	8	1	1	2	2	0	1
**10**	24	21	7	9	0	0	0	0	0	1
**11**	14	13	5	7	0	0	4	3	0	0
**12**	19	17	11	13	0	0	0	0	0	0
**13**	21	19	6	7	0	0	1	1	1	2

**Table 3:** Percentages of correlation by type of white blood cell.

correlation percentage	Lymphocyte	Neutrophil	Basophil	Monocyte	Eosinophilic
	93.42%	79.52%	100%	97.37%	73.07%

**Table 4:** Comparison between systems semiautomatic versus developed algorithm.

**Author**	**Techniques**	**Results**
[7]	Segmentation by OTSU and classification by neural networks.	Average performance of 65% and 95% after training.

[12]	Algorithm based on gram-Schmidt orthogonalization.	Average performance of 85.4%.

[11]	Iterative method of increasing region.	Effectiveness was obtained to identify the cells of 76.47% for Basophils, 95.5% for neutrophils.

[17]	Contrast adjustment in RGB and complex-value neural networks.	To precision in complex value of 99.3% and 97.5% in real value was obtained.

[18]	Image segmentation with contrast adjustment and filtering in grayscale.	An accuracy of 80.04%, 69.3%, 86.3%, 80.3% and 83.8% was obtained for basophils, eosinophils, monocytes, neutrophils and lymphocytes.

[19]	Classification by PCA and Dendrodendritic.	The average efficiency of the process was 77.2%.

[20]	The overlapped Detection of red blood cells in microscopic images of blood smear.	Sensitivity and specificity percentages were obtained higher than 96%

[21]	Classification of different types of white blood cells by global threshold and features geometrics.	Percentages of classification were obtained higher than 98%, 92% and 95% for lymphocyte, monocyte and neutrophil respectively.

[22]	Leukocyte nucleus segmentation and recognition by K-Means clustering.	Was obtained to precision of 98% for Basophil, 98% Eosinophil, 84.3% 93.3% Lymphocyte, monocyte and neutrophil 81.3.

[23]	Leukocytes Classification In Blood Smear by support vector machines (SVM).	Was obtained to accuracy of 98.5%, 99.9% Neutrophil for Eosinophil, 98.8% 93.7% Lymphocyte and Monocyte.

[16]	WBC Segmentation and Classification by Fuzzy C-Mean.	The accuracy of the process was 91% for the 5 types of cells.

**Proposed System**	Classification of cells by networks of Gaussian radial basis functions (RBFN) and morphological descriptors.	Was obtained to 100% accuracy of 73.07%, 93.42%, 97.37% and 79.52% for Basophiles, Eosinophil's, lymphocytes, monocytes and neutrophils respectively and 98.2%.

## Data Availability

The data used to support the findings of this study are available from the corresponding author upon request.
